# Effect of Thyroxine Replacement Therapy on Serum Maresin 1 and NF-kB Levels in Patients with Hashimoto Thyroiditis

**DOI:** 10.3390/diagnostics15101248

**Published:** 2025-05-14

**Authors:** Meltem Yardim, Levent Deniz, Mehmet Akif Saltabas, Nilufer Celik

**Affiliations:** 1 Department of Medical Biochemistry, Yerkoy State Hospital, 66900 Yozgat, Türkiye; 2 Department of Medical Biochemistry, University of Health Sciences, Istanbul Training and Research Hospital, 34098 Istanbul, Türkiye; levent.deniz33@gmail.com; 3 Department of Internal Medicine, Yerkoy State Hospital, 66900 Yozgat, Türkiye; sltbs66@gmail.com; 4 Department of Medical Biochemistry, Dr. Behcet Uz Children’s Hospital, 35210 Izmir, Türkiye; nilufercelik35@hotmail.com

**Keywords:** Hashimoto’s thyroiditis, NF-kB, Maresin 1, TPOAb, thyroxine replacement

## Abstract

**Background/Objectives:** This study aimed to investigate the effects of thyroxine replacement therapy (TRT) on serum Maresin 1 and nuclear factor kappa beta (NF-kB) levels in patients with Hashimoto’s thyroiditis (HT). **Methods:** A total of 90 patients were included in this study, 60 with HT and 30 without. Patients in the HT group were divided into two groups according to whether they received TRT. Group 1 included 30 patients who underwent TRT, and Group 2 comprised 30 patients who were newly diagnosed with HT, either euthyroid or hypothyroid. The analysis included serum levels of thyroid-stimulating hormone (TSH), free thyroxine (FT4), free triiodothyronine (FT3), thyroid peroxidase antibody (TPOAb), Maresin 1, and NF-kB. **Results:** The serum NF-kB level in the TRT group was significantly higher than that in the control and non-TRT groups. In the subgroup analysis of patients who did not receive TRT, the serum NF-kB level in euthyroid patients was significantly lower than that in hypothyroid patients. Maresin 1 levels in the control group were significantly higher than those in patients who did and did not receive TRT. The serum Maresin 1 level in the TRT group was significantly lower than that in the untreated group. Maresin 1 levels were higher in the euthyroid group than in the hypothyroid group. TPOAb levels were positively correlated with NF-kB and negatively correlated with Maresin 1. **Conclusions:** TRT maintains the euthyroid state in patients with HT, but may not contribute positively to the pro-anti-inflammatory balance in these patients.

## 1. Introduction

Hashimoto’s thyroiditis (HT) is the most common autoimmune endocrine pathology in humans and is thought to develop due to impaired self-tolerance of thyroid follicular cells [1]. HT is one of the most common causes of thyroid dysfunction and non-endemic goiter [2]. Loss of immune tolerance against thyroid cells leads to the destruction of the thyroid gland by lymphocytes and autoantibodies, thereby disrupting the synthesis and release of thyroid hormones. Although HT is an autoimmune disease, the mechanism triggering thyroid cell damage remains unclear. For the regular and adequate production of triiodothyronine (T3) and thyroxine (T4) hormones, a balanced redox state and subclinical inflammation in the thyroid gland are required. Reactive oxygen species or proinflammatory cytokine production beyond physiological limits may damage thyroid follicular cells [3,4,5]. To date, it has not been clearly established whether lymphocyte infiltration due to impaired immune tolerance causes thyroid follicle cell damage or whether increased proinflammatory cytokine production triggers damage to thyroid follicle cells.

Intracellular inflammation is critical for organ-specific functions and is regulated by the nuclear factor kappa-B (NF-kB) pathway. NF-kB, a transcription protein, maintains the intracellular inflammatory balance along with anti-inflammatory molecules [1]. While NF-kB is bound to inhibitory proteins in the cytoplasm under physiological conditions, it is activated under pathological conditions that disrupt the balance of proinflammatory cytokine release and stimulate the relevant target genes in the nucleus [6]. While balanced NF-kB expression regulates proliferation, differentiation, and apoptosis in thyroid cells, NF-kB overexpression may cause inflammatory and autoimmune pathologies [1,7,8,9]. Single-cell RNA sequencing and whole transcriptome analyses of thyroid tissue samples have shown that the NF-kB signaling pathway is differentially expressed in HT [10].

Although physiological inflammation is necessary to protect against harmful internal and external stimuli, excessive inflammation can lead to tissue damage [11]. Maresin 1 is a derivative of omega-3 fatty acids that are abundantly found in human macrophages. It exerts anti-inflammatory effects by inhibiting neutrophil migration and proinflammatory cytokine production [12]. Maresin 1 also provides immunomodulation by preventing Th1 and Th17 differentiation and enhancing regulatory T cell activation [13]. Maresin 1 increases phagocytosis and apoptosis via leucine-rich repeat-containing G protein-coupled receptor 6. Furthermore, Maresin 1 contributes to the resolution of inflammation by reducing the number of M1 macrophages and augmenting the number of M2 macrophages [14,15]. Although Maresin 1 inhibits proinflammatory cytokine release while increasing anti-inflammatory cytokine levels [16], no study has investigated the circulating levels of Maresin 1 in patients with HT. Considering the genetic, environmental, and autoimmune origins of HT [17], a deficiency in the dietary intake of omega-3 fatty acid derivatives, such as Maresin 1 [12], may cause HT by disrupting the inflammatory balance in the thyroid gland. This study aimed to investigate the effects of thyroxine replacement therapy (TRT) on serum Maresin 1 and NF-kB levels in patients with Hashimoto’s thyroiditis (HT). The relationships between serum NF-kB, Maresin 1, thyroid autoantibodies, and other demographic and laboratory parameters were also evaluated.

## 2. Materials and Methods

Based on the study by Wu et al. [18] and using G*Power (version 3.1.9.4; Heinrich-Heine-Universität Düsseldorf, Düsseldorf, Germany), an effect size of f = 0.56, an α significance level of 0.05, and (1-β) 0.80 of power were used to calculate the minimum sample size required to compare the three independent groups. The minimum total sample size was determined to be 36 patients.

In this cross-sectional study, a total of 90 patients, 60 previously or newly diagnosed HT patients, and 30 healthy controls, who visited the Yerkoy State Hospital Internal Medicine outpatient clinic between June 2023 and November 2023, were included. Ethics committee approval (date: 25 May 2023; Decision Number: 2017-KAEK-189_2023.05.25_01) was received from the Yozgat Bozok University Clinical Research Ethics Committee. Informed consent was obtained from all the participants.

HT patients were divided into two groups according to whether they received TRT. Group 1: Patients receiving TRT for Hashimoto’s thyroiditis (n = 30). Group 2: Newly diagnosed euthyroid or hypothyroid HT patients (n = 30). While 17 patients were newly diagnosed with euthyroid HT, there were 13 newly diagnosed with hypothyroid HT. Thus, Group 1 consisted of HT patients receiving TRT, while Group 2 consisted of HT patients not receiving TRT. Participants in the control group were selected from outpatients with no obvious endocrine pathologies. HT was diagnosed based on clinical symptoms and the presence of antithyroid antibodies (anti-thyroid peroxidase [TPOAb] against thyroid antigens). Diffuse and irregular thyroid enlargement and the presence of hypoechoic areas on ultrasonography were interpreted as indicators of HT. Patients with a large goiter compressing the cervical structures, suspicious thyroid cytology, malignancy, hematological disorders, previous thyroid surgery, endocrine or systemic diseases such as rheumatic disease, diabetes mellitus, active infection, systemic inflammatory disease, renal disease, liver failure, immunosuppressive treatment, and women with suspected pregnancy were excluded.

Blood samples were collected from all participants at 8 AM following an overnight fast. Although routine biochemical and hormonal parameters were measured on the same day, some serum samples were frozen for subsequent ELISA. Serum glucose, urea, creatinine, aspartate aminotransferase (AST), alanine aminotransferase (ALT), total cholesterol, high-density lipoprotein cholesterol (HDL-C), and triglyceride levels were measured using spectrophotometry with a DxC 700 AU chemistry analyzer (Beckman Coulter, Inc., Brea, CA, USA). Low-density lipoprotein cholesterol (LDL-C) levels were calculated using the Friedewald formula [19].

Serum thyroid-stimulating hormone (TSH), free thyroxine (FT4), free triiodothyronine (FT3), TPOAb, and 25-hydroxy vitamin D levels were measured by way of the chemiluminescence method using an Advia Centaur XP (Siemens Healthineers, Erlangen, Germany) device. The reference values for these tests were FT4 (0.89–1.76 ng/dL), FT3 (2.3–4.2 pg/mL), and TSH (0.35–5.5 μIU/mL), respectively. The reference ranges for TSH, FT4, and FT3 were set at the 2.5 to 97.5 percentiles. The sensitivity of the TSH kit was 0.008 µIU/mL, and the analytical measurement range was 0.008–150 µIU/mL. The sensitivity of the FT4 kit was 0.10 ng/dL, and the analytical measurement range was 0.10–12 ng/dL. The sensitivity of the FT3 kit was 0.2 pg/mL, and the analytical measurement range was 0.2–20 pg/mL. According to the method and reagent manufacturer’s guidelines, the threshold for the diagnosis of autoimmune thyroid disorder is 60 U/mL for anti-TPO, which is interpreted as positive for the presence of antibodies.

### 2.1. Measurement of Serum NF-kB and Maresin 1 with ELISA

Serum NF-kB and Maresin 1 levels were determined by NF-kB ELISA (Cusabio Biotech Co., Ltd., Catalog no. CSB-E12107h, Wuhan, China) and Maresin 1 ELISA (Sunred Biotechnology Company, Catalog no. 201-12-7339, Shanghai, China) and enzyme immunoassay principles. An automatic washer (Biochrom Anthos Fluido 2; Biochrom Ltd., Cambridge, UK) was used for plate washing. Absorbances were measured at a 450 nm wavelength using a CLARIOstar PLUS microplate reader (BMG Labtech, Ortenberg, Germany). The measurement range of the NF-kB kit was 0.312–20 ng/mL, and the sensitivity was 0.078 ng/mL. The measurement range of the Maresin 1 kit was 7.5–2000 pg/mL, and the sensitivity level was 7.247 pg/mL. The NF-kB kit demonstrated an intra-assay coefficient of variation (CV) below 8%, whereas the inter-assay CV was below 10%. For the Maresin 1 kit, the intra-assay CV was found to be less than 10%, with an inter-assay CV not exceeding 12%.

### 2.2. Statistical Analysis

SPSS version 27.0 (IBM Corp., Armonk, NY, USA) and GraphPad Prism 8.3.0 (GraphPad Software, San Diego, CA, USA) were used for the statistical analyses. The distribution patterns of the data were examined using the Shapiro–Wilk test. Parametric data are presented as the mean ± standard deviation, non-parametric data as the median (1st–3rd quartile), and categorical data as the number (percentage). One-way analysis of variance (ANOVA) was used to compare parametric variables, and the Kruskal–Wallis test was used to compare non-parametric variables. Categorical data analysis was conducted using either the Chi-square test or Fisher’s exact test, as appropriate for statistical comparison.

Pairwise comparisons for both one-way ANOVA and Kruskal–Wallis tests were performed using the Bonferroni correction, with the significance level set at 0.017 for comparisons among the three groups and 0.008 for comparisons among the four groups. The correlations between variables were examined using Spearman’s correlation analysis. A correlation matrix was created using the R software (v. 4.3.3) “corrplot” package [20]. Statistical significance was set at *p* < 0.05.

## 3. Results

**Figure 1:**
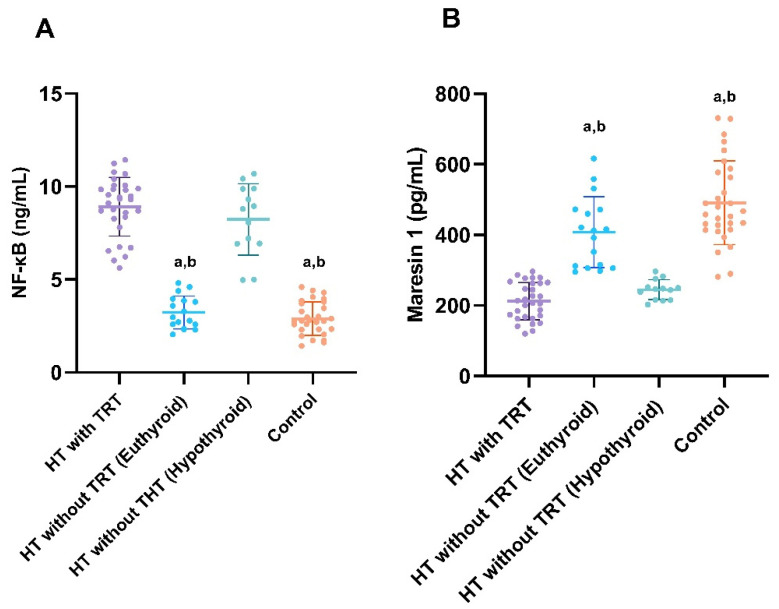
Graphical representation of serum NF-kB (**A**) and Maresin 1 levels (**B**) in the TRT-treated, non-treated, and control groups. ^a^ *p* < 0.001 vs. hypothyroid HT group; ^b^ *p* < 0.001 vs. HT plus THT group.

Of the 60 patients in the HT group, 51 (85.0%) were women and 9 (15.0%) were men. The control group consisted of 30 volunteer participants [24 (80.0%) women and 6 (20.0%) men] who were matched to the HT group with respect to age, body mass index (BMI), and sex. In the control group, Maresin 1 levels were significantly higher than those in the HT patients who did and did not receive TRT (*p* < 0.001 for each). Serum Maresin 1 levels in the TRT group were significantly lower than in the no-replacement group (*p* < 0.001). Serum TSH levels in Groups 1 and 2 were significantly higher than those in the control group (*p* = 0.007 and *p* < 0.001, respectively). TSH levels were similar in Groups 1 and 2. Serum FT3 and FT4 levels in Group 2 were significantly lower than in the control group (*p* = 0.006 and *p* = 0.012, respectively). TPOAb levels in Groups 1 and 2 were similar and higher than those in the control group (*p* < 0.001 for each). Serum vitamin D levels in Groups 1 and 2 were similar and significantly lower than those in the control group (*p* < 0.001 and *p* = 0.014, respectively). Other laboratory parameters in the HT and control groups were similar (Table 1). NF-kB levels in HT patients treated with TRT were significantly higher than those in the HT group without TRT (*p* < 0.001). Serum NF-kB levels of the HT group without TRT were significantly higher than those of the control group (*p* = 0.001) (Table 1). While NF-kB levels in the euthyroid group were similar to those in the control group and NF-kB levels in the hypothyroid group were similar to HT with TRT, NF-kB levels were significantly higher in both HT with TRT and hypothyroid HT without TRT compared to control and euthyroid HT without TRT (Figure 1A). While Maresin 1 levels in the euthyroid group were similar to those in the control group and Maresin 1 levels in the hypothyroid group were similar to HT with TRT, Maresin 1 levels were significantly lower in both HT with TRT and hypothyroid HT without TRT compared to control and euthyroid HT without TRT (Figure 1B). In the subgroup analysis of Group 2, serum TSH levels in the hypothyroid group were higher than those in the euthyroid group [8.34 (7.84–8.85) vs. 2.57 (2.11–3.93); *p* < 0.001]. In the subgroup analysis of Group 2, FT4 levels in the euthyroid group were significantly higher than those in the hypothyroid group [1.21 (1.03–1.35) vs. 0.68 (0.60–0.78); *p* < 0.001]. In the subgroup analysis of Group 2, the FT3 levels of the euthyroid patients were significantly higher than those of the hypothyroid group [3.18 (2.90–3.30) vs. 2.15 (2.04–2.29); *p* < 0.001].

The TPOAb levels in the euthyroid group were significantly lower than those in the hypothyroid group [279 (230–363) vs. 819 (775–961); *p* < 0.001)] (Table 2). In the HT group, there was a negative correlation between NF-kB and vitamin D (r = −0.795, *p* < 0.001), FT4 (r = −0.265, *p* = 0.041), FT3 (r = −0.319, *p* = 0.013), and HDL-C (r = −0.315, *p* = 0.014). NF-kB showed positive correlation with age (r = 0.283, *p* = 0.028) and TPOAb (r = 0.677, *p* < 0.001).

**Figure 2:**
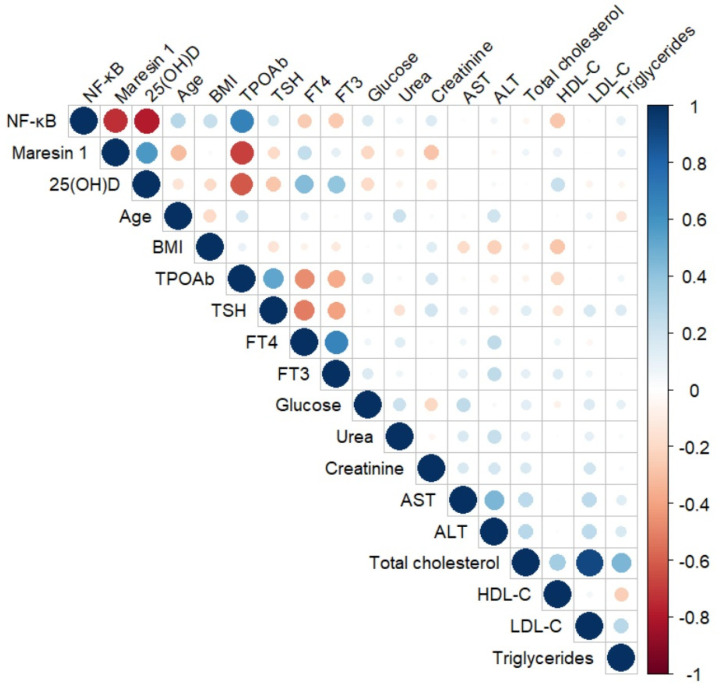
Correlation matrix showing the relationships between NF-kB and Maresin 1 levels and other parameters. Spearman’s rho test was used in the matrix drawing. The numbers ranging from −1 to +1 on the vertical axis represent Spearman’s rank correlation coefficients. Blue represents a positive correlation, and red represents a negative correlation. The color depth and size of the circles indicate the strength of correlation.

In the HT group, there was a negative correlation between Maresin 1 and NF-kB (r = −0.729, *p* < 0.001), age (r = −0.296, *p* = 0.022), TPOAb (r = −0.634, *p* < 0.001), glucose (r = −0.273, *p* = 0.035), and creatinine (r = −0.330, *p* = 0.010). Maresin 1 levels were positively correlated with vitamin D levels (r = 0.530, *p* < 0.001) (Table 3 and Figure 2).

## 4. Discussion

Serum Maresin 1 levels were significantly lower in HT patients who received levothyroxine than in those who did not receive replacement therapy but were euthyroid. Another important finding was that serum NF-kB levels in patients with HT who did not receive replacement therapy but were euthyroid and in the control group were lower than those in patients who received TRT or were hypothyroid HT patients without therapy. Serum NF-kB levels in the euthyroid group were significantly lower than in the hypothyroid group. However, the levels of Maresin 1 and NF-kB markers were similar between the hypothyroid group without TRT and the group receiving TRT. Therefore, we suggest that TRT can improve thyroid function without affecting autoimmune and inflammatory changes in the thyroid tissue. The dogma that organisms show self-tolerance to their antigens ended with the detection of antibody formation in animals injected with pure thyroglobulin [21]. Subsequent experiments revealed that repeated thyroglobulin injections triggered both antibody formation and inflammation. The detection of thyroglobulin antibodies in patients with HT in subsequent clinical studies has introduced the concept of autoimmune thyroiditis, an immune and inflammatory pathology, in the current literature [22]. The relationship between NF-kB and HT, the primary pathway for regulating the inflammatory balance in the thyroid gland, has been extensively studied by many researchers [1,10]. In parallel, the association between NF-kB overexpression and HT in thyroid follicular cells has been previously demonstrated [8]. The current study provides the first clinical data comparing serum NF-kB levels in patients with HT receiving thyroid hormone replacement therapy with those in newly diagnosed patients with HT not yet receiving replacement therapy. The fact that serum NF-kB levels in HT patients administered levothyroxine replacement therapy are higher than those in patients who have not received replacement therapy suggests that autoimmune and inflammatory reactions that cause damage to the thyroid glands continue despite levothyroxine therapy. In agreement with this, in addition to genes related to Th1, Th2, and Th17 cells, NF-kB signaling genes are differentially expressed in the thyroid follicle cells of patients with HT [10]. As Th17 cells differentiate through NF-kB, causing thyroid antigens to be perceived as foreign [9,10], high serum NF-kB levels may be important evidence that follicular destruction continues despite levothyroxine treatment.

Although levothyroxine replacement therapy regulates many genes in leukocytes and platelets [23], its effects on autoimmune and inflammatory pathways in thyroid tissues have not been fully elucidated. Analysis of pro- and anti-inflammatory molecules at different levothyroxine doses and repeated serum sampling will provide clearer results on how TRT affects the inflammatory process in patients with HT.

The fact that the serum NF-kB levels of the euthyroid group that did not receive synthetic levothyroxine treatment were lower than those in the replacement group supports the theory that synthetic levothyroxine has a lower protective effect on thyroid tissue from the destructive effects of autoimmune and inflammatory reactions than physiological levothyroxine. The serum NF-kB levels of hypothyroid patients with HT who did not receive levothyroxine treatment were similar to those in the group that received replacement therapy, which may support the idea that levothyroxine replacement does not change the autoimmune process in the thyroid gland, despite its many physiological effects. Consistent with this, the failure to achieve complete remission in specific laboratory findings and clinical complaints in many patients despite achieving biochemical euthyroidism supports the notion that synthetic levothyroxine does not treat autoimmune and inflammatory reactions in thyroid tissue [23,24,25]. However, since HT is accompanied by other systemic autoimmune and inflammatory diseases in 20% of cases, it is thought that the increase in serum NF-kB levels, despite levothyroxine treatment, may not be due to HT alone [26]. The loss of immune tolerance induced by genetic predisposition and environmental factors is the basic mechanism that initiates follicle cell destruction, which is mediated by chemokines and proinflammatory cytokines [26].

Despite levothyroxine treatment, high NF-kB transcript levels may lead to gradual atrophy and fibrosis in the thyroid tissue, leading to thyroid gland damage [1,7,8,9,22]. The positive association between NF-kB and TPOAb levels supports this hypothesis. To prevent NF-kB-mediated gland damage, different anti-inflammatory mechanisms come into play and try to maintain the pro- and anti-inflammatory balance in the thyroid gland [10,26]. Low levels of vitamin D, antioxidants, omega-3 fatty acids, and selenium in patients with HT are thought to worsen the disease course by increasing oxidative stress and proinflammation [27].

The fact that the serum vitamin D levels in the HT group were lower than those in the control group supports this hypothesis. However, the fact that serum 25(OH)D levels are reported to be unrelated to thyroid function and antithyroid antibodies in autoimmune troiditis weakens our hypothesis of a possible relationship between vitamin D and HT [28]. Another molecule recommended as a replacement for HT is omega-3 fatty acids [27]. Maresin 1, an omega-3 fatty acid derivative, is a powerful anti-inflammatory molecule and its level in HT patients has not been investigated. The negative correlation between serum NF-kB and Maresin 1 levels in the HT group supports the disruption of the pro-anti-inflammatory balance in thyroid follicles. Similarly, the negative correlation between Maresin 1 and TPOAb suggests that Maresin 1 may prevent the formation of anti-thyroid antibodies (ATAs). The fact that Maresin 1 levels were lower in the TRT group than in the group without replacement therapy suggests that serum Maresin 1 levels were low, independent of TRT. Consistent with our results, decreased serum levels of resolvin E1, an omega-3 polyunsaturated fatty acid-derived anti-inflammatory molecule, in HT support the existence of inadequate anti-inflammatory molecule expression [29]. Consequently, failure to balance NF-kB overexpression due to low Maresin 1 levels in the HT group may have caused continued damage to the thyroid tissue and failed to reduce TPOAb levels. The fact that Maresin 1 levels remained low in the TRT group suggests that levotroxine does not have a clear stimulatory effect on the anti-inflammatory pathways in the thyroid gland. In line with this, it has been reported that although there was an improvement in inflammation in patients with HT who were given anti-inflammatory supplements in addition to a gluten-free diet, there was no significant change in the levels of anti-thyroid antibodies [30]. This study is of clinical importance because it investigated the effects of TRT on redox balance using new markers and provided usable clinical data. With more participants, rarer differences could be detected. However, a power analysis confirmed that the number of participants was sufficient for the statistical evaluation. However, the study of only two markers of redox balance is a limitation in making clear statements.

Our findings may recommend the re-evaluation of some clinical practices in HT patients. The fact that thyroid gland pathology is not positively affected by treatment in HT patients receiving levothyroxine replacement therapy indicates that antithyroid antibody levels will continue to be high in a clinically stable patient. Since replacement therapy does not correct the autoimmune and inflammatory processes in the thyroid gland, high antibody levels can be detected for a long time. Therefore, replacement duration and dose should not be determined according to serum TPOAb levels. If the clinician deems it appropriate, anti-inflammatory and immune modulatory therapy can be added in addition to replacement therapy.

## 5. Conclusions

This study is important in terms of showing that levothyroxine treatment in HT patients replaces deficient thyroid hormones rather than correcting the inflammatory pathology in the thyroid gland. Levothyroxine replacement therapy is critical for treating hypothyroidism in patients with HT-induced autoimmune thyroid diseases. In the current study, we aimed to determine whether TRT has a corrective effect on the basic pathology of thyroid lesions. Comprehensive studies are needed to evaluate multiple redox markers in both serum and thyroid tissue according to the given drug doses. Despite the small sample size, our study is clinically important because it shows the relationship between serum Maresin 1 and NF-kB in patients with HT. Disturbance to the proinflammatory balance in favor of inflammation in the thyroid tissue of patients with HT leads to persistent tissue damage. Since levothyroxine replacement therapy does not reduce NF-kB expression or increase circulating levels of anti-inflammatory molecules such as Maresin 1, we can assume that it does not have a net effect on pro- and anti-inflammatory balance in HT patients. TRT replaces deficient T3 and T4 levels rather than having a curative effect on autoimmune and inflammatory diseases of the thyroid gland.

## Figures and Tables

**Table 1:** Analysis of the demographic and biochemical parameters of the patients in the HT group who did and did not receive thyroxine replacement therapy, and the control group.

Parameter.	HT Plus TRT (Group 1; n = 30)	HT Without TRT (Group 2; n = 30)	Control Group (n = 30)	*p*-Value
Age (years)	31.6 ± 4.00	29.3 ± 3.96	30.7 ± 4.92	0.114 ^‡^
Gender (male/female)	5 (16.7%) 25 (83.3%)	4 (13.3%) 26 (86.7%)	6 (20.0%) 24 (80.0%)	0.787 ^‡‡‡^
Body Mass Index (kg/m^2^)	24.9 (24.0–25.8)	24.9 (23.6–26.1)	24.3 (23.0–25.3)	0.380 ^‡‡^
NF-κB (ng/mL)	9.31 (8.27–9.88) **^, ##^	4.48 (2.78–8.05) *	2.77 (2.29–3.70)	<0.001 ^‡‡^
Maresin 1 (pg/mL)	210 (168–263) **^, ##^	302 (248–416) **	462 (415–577)	<0.001 ^‡‡^
25(OH)D (ng/mL)	13.6 ± 3.51 **	15.9 ± 5.54 *	20.6 ± 6.63	<0.001 ^‡^
TPOAb (U/mL)	700 (440–845) **	706 (250–805) **	5.54 (3.96–10.7)	<0.001 ^‡‡^
TSH (µIU/mL)	3.46 (2.53–5.80) *	5.95 (2.51–8.30) **	2.54 (1.66–2.89)	<0.001 ^‡‡^
FT4 (ng/dL)	1.11 ± 0.14	0.96 ± 0.31 *	1.16 ± 0.17	0.002 ^‡^
FT3 (pg/mL)	2.95 ± 0.41	2.67 ± 0.55 *	3.06 ± 0.34	0.004 ^‡^
Glucose (mg/dL)	95.0 (90.0–100)	92.0 (87.0–99.0)	91.5 (86.0–98.0)	0.201 ^‡‡^
Urea (mg/dL)	26.2 ± 6.78	26.0 ± 8.21	24.1 ± 7.40	0.500 ^‡^
Creatinine (mg/dL)	0.70 (0.60–0.80)	0.65 (0.60–0.70)	0.61 (0.60–0.70)	0.182 ^‡‡^
ALT (IU/L)	15.0 (13.0–23.0)	14.5 (12.0–19.0)	17.0 (15.0–20.0)	0.175 ^‡‡^
AST (IU/L)	20.0 (18.0–24.0)	21.5 (17.0–24.0)	19.0 (17.0–24.0)	0.935 ^‡‡^
TC (mg/dL)	178 (148–205)	174 (158–195)	179 (161–207)	0.999 ^‡‡^
HDL-C (mg/dL)	52.3 ± 16.1	55.7 ± 14.6	58.1 ± 15.4	0.350 ^‡^
LDL-C (mg/dL)	99.5 (88.0–127)	97.5 (82.0–114)	101 (80.0–127)	0.806 ^‡‡^
Triglycerides (mg/dL)	108 (81.0–152)	101 (65.0–135)	93.5 (71.0–137)	0.694 ^‡‡^

^‡^: One-way ANOVA test, ^‡‡^: Kruskal–Wallis test, ^‡‡‡^: Chi-square test. HT: Hashimoto’s thyroiditis, TRT: thyroxine replacement therapy, NF-κB: nuclear factor kappa B, 25(OH)D: 25-hydroxy vitamin D, TPOAb: anti-thyroperoxidase antibody, TSH: thyroid-stimulating hormone, FT4: free thyroxine, FT3: free triiodothyronine, ALT: alanine aminotransferase, AST: aspartate aminotransferase, TC: total cholesterol, HDL-C: high-density lipoprotein cholesterol, LDL-C: low-density lipoprotein cholesterol. * *p* < 0.017, ** *p* < 0.001 vs. control; ^##^ *p* < 0.001 vs. HT without TRT group.

**Table 2:** Comparison of thyroid function tests, NF-κB, and Maresin 1 levels between groups.

	HT with TRT (Group 1; n = 30)	HT Without TRT (Group 2; n = 30)		Control (n = 30)	*p*-Value
		Euthyroid HT (n = 17)	Hypothyroid HT (n = 13)		
TSH (µIU/mL)	3.46 (2.53–5.80) **^d^**	2.57 (2.11–3.93) **^a^**	8.34 (7.84–8.85)	2.54 (1.66–2.89) **^a^**^,**e**^	*p* < 0.001 ^‡‡^
FT4 (ng/dL)	1.11 (1.02–1.19) **^a^**	1.21 (1.03–1.35) **^a^**	0.68 (0.60–0.78)	1.14 (1.05–1.24) **^a^**	*p* < 0.001 ^‡‡^
FT3 (pg/mL)	3.04 (2.81–3.25) **^a^**	3.18 (2.90–3.30) **^a^**	2.15 (2.04–2.29)	3.10 (2.89–3.34) **^a^**	*p* < 0.001 ^‡‡^
TPOAb (U/mL)	700 (440–845)	279 (230–363) **^a^**^,**e**^	819 (775–961)	5.54 (3.96–10.7) **^a^**^,**b**,**c**^	*p* < 0.001 ^‡‡^
NF-κB (ng/mL)	8.91 ± 1.57	3.23 ± 0.88 **^a^**^,**b**^	8.23 ± 1.91	2.88 ± 0.90 **^a^**^,**b**^	*p* < 0.001 ^‡^
Maresin 1 (pg/mL)	212 ± 52.5	408 ± 99.8 **^a^**^,**b**^	245 ± 28.4	491 ± 119 **^a^**^,**b**^	*p* < 0.001 ^‡^

^‡^: One-way ANOVA test, ^‡‡^: Kruskal–Wallis test. HT: Hashimoto’s thyroiditis, TRT: thyroxine replacement therapy, TSH: thyroid-stimulating hormone, FT4: free thyroxine, FT3: free triiodothyronine, TPOAb: anti-thyroperoxidase antibody, NF-κB: nuclear factor kappa B. **^a^***p* < 0.001 vs. hypothyroid HT group; **^b^** *p* < 0.001 vs. HT with TRT group; **^c^** *p* < 0.001 vs. euthyroid HT group; **^d^** *p* < 0.008 vs. hypothyroid HT group; **^e^** *p <* 0.008 vs. HT with TRT group.

**Table 3:** Correlation between NF-κB, Maresin 1, and other variables in patients with Hashimoto’s thyroiditis.

Parameters		NF-κB	Maresin 1
NF-κB (ng/mL)	r	-	−0.729
	p	-	<0.001
25(OH)D (ng/mL)	r	−0.795	0.530
	p	<0.001	<0.001
Age (years)	r	0.283	−0.296
	p	0.028	0.022
Body Mass Index (kg/m^2^)	r	0.199	−0.030
	p	0.127	0.819
TPOAb (U/mL)	r	0.677	−0.634
	p	<0.001	<0.001
TSH (µIU/mL)	r	0.174	−0.144
	p	0.184	0.272
FT4 (ng/dL)	r	−0.265	0.217
	p	0.041	0.096
FT3 (pg/mL)	r	−0.319	0.178
	p	0.013	0.173
Glucose (mg/dL)	r	0.139	−0.273
	p	0.288	0.035
Urea (mg/dL)	r	0.115	−0.138
	p	0.383	0.291
Creatinine (mg/dL)	r	0.160	−0.330
	p	0.221	0.010
AST (IU/L)	r	0.003	−0.129
	p	0.979	0.326
ALT (IU/L)	r	0.038	−0.111
	p	0.773	0.397
TC (mg/dL)	r	−0.034	0.078
	p	0.795	0.556
HDL-C (mg/dL)	r	−0.315	0.126
	p	0.014	0.339
LDL-C (mg/dL)	r	0.060	−0.023
	p	0.649	0.862
Triglycerides (mg/dL)	r	0.124	0.046
	p	0.345	0.725

r: correlation coefficient, p: *p*-value, statistical test: Spearman’s correlation. Statistically significant results are bolded. NF-κB: nuclear factor kappa B, 25(OH)D: 25-hydroxy vitamin D, TPOAb: anti-thyroperoxidase antibody, TSH: thyroid-stimulating hormone, FT4: free thyroxine, FT3: free triiodothyronine, ALT: alanine aminotransferase, AST: aspartate aminotransferase, TC: total cholesterol, HDL-C: high-density lipoprotein cholesterol, LDL-C: low-density lipoprotein cholesterol.

## Data Availability

All data pertaining to this study are presented in the article and there is no additional data to present.
